# Diaphragm Muscle Atrophy Contributes to Low Physical Capacity in COVID-19 Survivors

**DOI:** 10.3390/life14091117

**Published:** 2024-09-05

**Authors:** Janusz Kocjan, Mateusz Rydel, Jan Szczegielniak, Katarzyna Bogacz, Mariusz Adamek

**Affiliations:** 1 Faculty of Medicine with Dentistry Division, Department of Thoracic Surgery, Medical University of Silesia, 40-055 Katowice, Polandm.adamek@e.pl (M.A.); 2 Faculty of Physical Education and Physiotherapy, Department of Physical Education and Physiotherapy, Opole University of Technology, Prószkowska 76, 45-758 Opole, Poland; 3 Faculty of Health Sciences with Institute of Maritime and Tropical Medicine, Department of Radiology, Medical University of Gdansk, 80-210 Gdansk, Poland

**Keywords:** diaphragm, COVID-19, SARS-CoV-2, pulmonary rehabilitation

## Abstract

Fatigue and dyspnea are the most commonly reported long-term complaints in individuals previously infected with SARS-CoV-2. This study aimed to comprehensively evaluate diaphragm muscle function in post-COVID-19 patients and investigate whether potential diaphragm dysfunction contributes to physical functioning impairment. A total of 46 patients who qualified for pulmonary rehabilitation were examined. Diaphragm muscle function parameters were evaluated using ultrasonography, while the severity of dyspnea, aerobic capacity, and the amount of energy used by the body during physical activity were assessed using the six-minute walk test, mMRC scale, and Metabolic Equivalent Task (MET), respectively. We identified that 69.5% of patients had diaphragm atrophy and 6.5% had diaphragm paralysis. The percentage of atrophy was not related to age, gender, BMI, oxygen therapy usage during the COVID-19 infection course, and disease severity. Patients who experienced cough, fever, and no loss of smell during the COVID-19 course had significantly greater diaphragm inspiratory thickness values, while patients with cough and no smell disorders had a significantly lower percentage of diaphragm atrophy. Diaphragm functional parameters were strongly associated with selected variables of exercise tolerance, such as distance in the six-minute walk test, oxygen saturation levels, fatigue, and exertion on the Borg scale. In conclusion, diaphragm muscle dysfunction is a serious long-term post-COVID-19 consequence and can be viewed as a major contributing factor to prolonged functional impairments.

## 1. Introduction

The diaphragm is a crucial muscle for ventilation and the gas exchange process. The movement of this muscle creates morphological and functional alterations in the thoracic and abdominal cavities. Caudal and cranial movements of the diaphragm are associated with the inspiration and expiration phases of respiration, respectively. Proper functioning of the diaphragm is also important for expulsive actions, such as coughing and sneezing, which are necessary for clearing the airways and maintaining their patency [1,2].

Similar to any other skeletal muscles in the human body, the diaphragm can also be subject to dysfunction. Numerous conditions (i.e., congenital defects, hernias, traumas, cardiothoracic surgeries, and thoracic or abdominal pathologies) or systemic diseases (i.e., muscular dystrophies, sarcoidosis, and amyloidosis) can directly affect the diaphragm, leading to its weakness or/and paralysis, and consequently reduce breathing capability and efficient lung aeration [3,4]. Previous studies have reported the presence of diaphragm disorders over the course of viral infections, such as a human immunodeficiency virus [5,6], poliovirus [7], West Nile virus [8], dengue virus [9], herpes zoster virus [10], and Zika virus [11].

SARS-CoV-2 is an RNA virus that has become a major public health threat. The clinical manifestations of COVID-19 infection vary, ranging from asymptomatic to mild and moderate symptoms, to a serious clinical picture requiring intensive care [12]. Many studies focused on various aspects of this pandemic have been developed, including the nature and prevalence of post-COVID-19 symptoms. According to the literature, a large number of post-COVID-19 patients suffer from persistent dyspnea and fatigue, accompanied by some or much difficulty in walking, lifting, carrying, walking upstairs, and walking fast, even months after the onset of the disease [13]. These first two respiratory symptoms are also among the top five post-COVID-19 symptoms [14,15]. According to Cesanelli et al., COVID-19 can significantly affect diaphragm muscle function through a combination of direct and indirect mechanisms. First, the severe inflammatory response triggered by SARS-CoV-2 infection, characterized by the release of proinflammatory cytokines, can lead to weakness, fatigue, and diaphragm muscle atrophy. Additionally, patients with severe COVID-19 often require prolonged mechanical ventilation, which can result in ventilator-induced diaphragm dysfunction (VIDD) and lead to a loss of contractile strength due to diaphragm inactivity during assisted respiration. Finally, SARS-CoV-2 has been detected in various tissues, including muscle tissue. Direct viral invasion into muscle tissue can cause viral-induced myopathy, contributing to both structural and functional alterations in the diaphragm [16]. Therefore, diaphragm muscle dysfunction may be one of the factors contributing to prolonged functional impairments. Hence, to fill the gap in existing literature, we designed this study to identify and clarify the possible impact of SARS-CoV-2 infection on diaphragm muscle function.

The purpose of this study was to assess diaphragm muscle function among patients recovered from SARS-CoV-19 infection. We also strove to (I) estimate the prevalence of diaphragm muscle dysfunction, (II) establish causal relations between selected clinical variables and the potential risk of diaphragm dysfunction, and (III) determine the relationship between diaphragm functional parameters and physical functional capacity.

## 2. Materials and Methods

### 2.1. Study Design and Settings

A retrospective double-blind study was conducted at the Physical Therapy Department at the Ministry of Internal Affairs and Administration’s Specialist Hospital in Glucholazy (Poland). The study was approved by the Institutional Review Board and the Ethics Committee of Silesian Medical University (Decision Number: PCN/0022/KB1/09/21). All procedures performed in this study were in accordance with the ethical standards and with the 1964 Helsinki Declaration and its later amendments. Informed consent was obtained from all individual participants involved in the study. The authors had no access to information that could identify individual participants during or after data collection. The double-blind procedure referred to the individuals performing the measurements. The ultrasonographer had no access to the results of the survey and the 6-min walk test. The staff conducting the 6-min walk test with patients were not aware of the diaphragm ultrasound measurement values. Additionally, the staff who collected surveys had no knowledge of the 6-min walk test results or the ultrasound diaphragm parameters.

### 2.2. Participants

A total of 114 participants, who had recovered from COVID-19 infection and qualified for a three-week post-COVID-19 rehabilitation program, were recruited between 1 March and 30 July 2021. COVID-19 severity for each patient was classified into four groups (mild, moderate, severe, and critical) based on the Guidance for Coronavirus Disease 2019 (7th edition) released by the National Health Commission of China [17]. The determination of the infecting viral lineage was not performed, but considering the time of illness, the clinical course of the disease, and the epidemiological situation in our country, patients were likely infected with the Alpha lineage (B.1.1.7) of COVID-19, according to GISAID (Global Initiative on Sharing All Influenza Data) [18].

Patients were eligible for enrollment if they had at least one symptom during the infection course (coughing, fever, dyspnea, and loss of smell or taste) and if SARS-CoV-2 infection was confirmed by a RT-PCR (real-time reverse polymerase chain reaction) test of nasopharyngeal swabs. Participants were excluded from analysis for one or more of the following reasons: previous thoracic or abdominal surgery, presence of a disease that interferes with diaphragm function, previous participation in post-COVID rehabilitation, difficulty obtaining a clear view of the diaphragm upon ultrasound imaging, not complying with commands concerning breathing maneuvers during examination, presence of balance disorders that impeded ultrasound measurements in a standing position, or inability to participate in or finish the 6-min walk test.

### 2.3. Diaphragm Muscle Ultrasound

Diaphragm muscle function was evaluated using ultrasonography. The right and left hemidiaphragm were visualized through the liver and spleen window, respectively, in the zone of apposition area as a three-layer structure consisting of hyperechogenic lines of pleural and peritoneal membranes and the hypoechogenic layer of the muscle itself. A high-frequency (7–13 MHz) linear probe set to B mode was placed perpendicular to the chest wall in the midaxillary line, between the 8th and 10th intercostal spaces. In the case of the “lung curtain sign” artifact when the lung obliterated the muscle’s image, the operator moved the probe toward the anterior axillary line or in the caudal direction to the next intercostal space [19,20].

All measurements were performed with the Sonoscape E1 ultrasound machine before beginning the physiotherapy process. Prior to measurements, the participants were given verbal instructions about breathing maneuvers to perform. Based on the literature review mentioned in the Introduction Section, which suggested that patients who contracted COVID-19 have respiratory problems, especially during activity, we decided to perform ultrasound not only in the supine but also in the unsupported standing position. Ultrasonography examinations were performed by a well-trained specialist, who was blinded to the clinical data. Once a clear image of the diaphragm was obtained, the participants were asked to take a maximal inspiration (ThIns) and then exhale to end expiration (ThExp). On each frozen B-mode image, the diaphragm thickness was estimated as the vertical distance between pleural and peritoneal lines. Three images for each position and for each side were collected, and the average value was included in the statistical analysis [19,20].

From the obtained diaphragm measurements, we calculated the following diaphragm function indexes: (1) The diaphragm thickness fraction (DTF), reflecting muscle effort, was computed using the formula: DTF = (thickness at end inspiration—thickness at end expiration)/thickness at end expiration × 100%, and a value less than 20% was interpreted as hemidiaphragm paralysis. (2) The diaphragm thickening ratio (DTR), assessing the quality of muscle function, was calculated as the thickness at end inspiration divided by the thickness at end expiration. (3) An expiratory thickness less than 2 mm was assumed as a cutoff value for diaphragm muscle atrophy diagnosis. (4) The “DTR module” and “DTF module” for supine and standing positions were calculated to perform correlation analysis between the DTR index and DTF index with clinical variables. We used the following formulas: module DTR = (left ThIns/left ThExp)/(right ThIns/right ThExp), and module DTF = (left DTF + right DTF)/2 [19,20].

From all available diaphragm imaging techniques, we chose ultrasound examination as a non-invasive, valid, and reliable tool for assessing muscle action. According to previous studies, sonography results correlate with the size of the diaphragm compound muscle action potential in response to phrenic nerve stimulation [21], as well as with inspiratory muscle strength and pulmonary function [3,22]. Ultrasound techniques have also been shown to outperform traditional techniques, such as fluoroscopy, in diagnosing diaphragm dysfunction [23].

### 2.4. The mMRC (Modified Medical Research Council) Scale

The mMRC scale is a self-rating tool to assess the severity of dyspnea during daily living activities. The questionnaire consists of 5 statements evaluating the degree of dyspnea, graded on a scale from 0 (no breathlessness except on strenuous exercise) to 4 (complete incapacity to leave the house or breathless when dressing or undressing) [24].

### 2.5. The Six-Minute Walk Test

The 6-min walk test is a sub-maximal exercise test used to assess aerobic capacity and endurance. The patients were instructed that the purpose is to walk as far as possible during 6 min. In the present study, the test was performed using the updated methodology specified by the American Thoracic Society and European Respiratory Society. The primary outcome measure was the 6-min walk distance. Heart rate, blood pressure, and oxygen saturation were also collected at the beginning and at the end of the 6-min walk test [25].

### 2.6. Rating of Perceived Exertion: The Borg Scale

The Borg Rating of Perceived Exertion (RPE) scale is a subjective, self-assessed measure of exercise intensity, based on an individual’s perception of physical exertion, without relying on physiological parameters, such as peak oxygen uptake, heart rate, or lactate levels. Patients were instructed to rate their exertion after completing the 6-min walk test by selecting a number from the scale that best reflects their overall effort. A rating of 6 represents no exertion (i.e., rest), while a rating of 20 represents maximal exertion (i.e., the most strenuous exercise performed) [26].

### 2.7. MET (The Metabolic Equivalent) Score

The Metabolic Equivalent Task (MET) concept represents a procedure for expressing the energy cost of physical activities as a multiple of the resting metabolic rate. One MET is represented in the literature by the oxygen consumption (VO_2_) at rest in a sitting position by a person weighing 70 kg, approximately 3.5 mL/kg/min. The energy expenditure expressed in METs is commonly adapted from statistically derived published tables, representing the number of times by which the resting metabolism was multiplied during an activity. In the present study, energy expenditure expressed in MET units was calculated after performing the 6-min walk test (6-MWT) based on the covered distance, which was converted into walking speed using the following equation: distance × 10/1000. The previously existing formula for calculating MET based on the 6-MWT was intended for people with severe limitations in exercise capacity, excluding patients who covered a distance of more than 300 m. The modified formula has been tested and proven in COPD patients and has been used for patients after COVID-19 with limited exercise capacity [27].

### 2.8. Statistical Analysis

The sociodemographic data, as well as clinical signs and patients’ symptoms over the COVID-19 course, were collected from available medical records and a separate questionnaire. We used an Excel sheet to gather all variables. Descriptive data are expressed as the mean ± standard deviation (including 95% confidence interval), and/or percentages. Comparisons between two groups for categorical variables were performed using the χ^2^ test or Fisher’s exact test, while comparisons for continuous variables were conducted using Student’s *t*-test or the Mann–Whitney U test. Correlation analysis was carried out using Spearman’s correlation test. Data analysis was performed using PQStat software version 1.8.6.102 for Windows. Here, *p*-values lower than 0.05 were considered statistically significant.

## 3. Results

**Figure 1:**
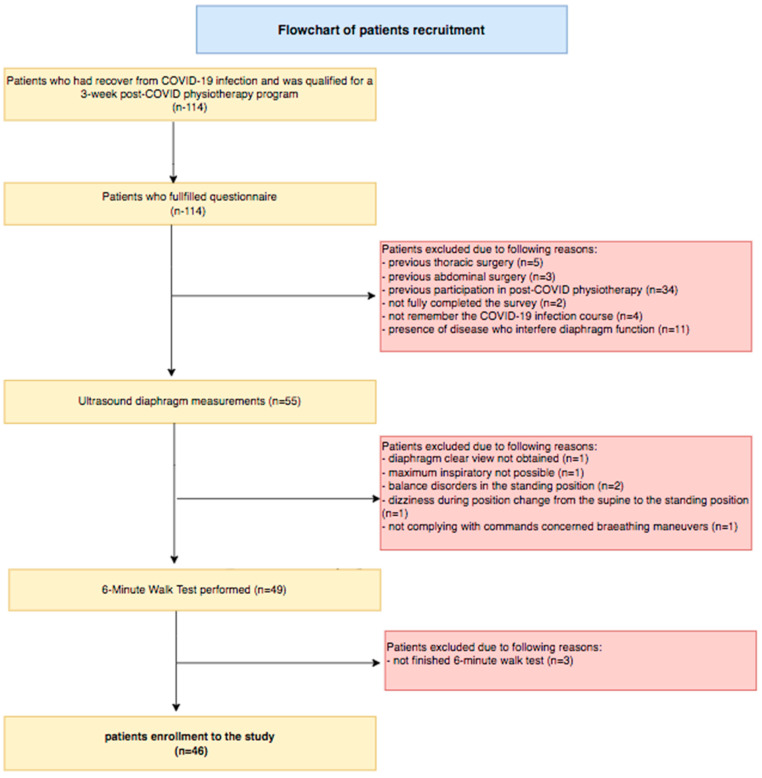
Flowchart of patient recruitment.

Forty-six patients were enrolled in the study. The flowchart of the patient recruitment process is presented in Figure 1.

The demographic data of the study participants and their clinical characteristics are shown in Table 1. There were no differences between subgroups in terms of gender composition (*p* = 0.382), age (*p* = 0.069), BMI (*p* = 0.196), saturation (*p* = 0.403), oxygen therapy use (*p* = 0.461), and mMRC scale (*p* = 0.113). None of the patients had the disease twice before participation in this study.

Descriptive statistics of diaphragm functional parameters are presented in Table 2. The vast majority of patients had worse inspiratory (right dome: 91.3%, n = 42; left dome: 86.9%, n = 40) and expiratory (right dome: 82.6%, n = 38; left dome: 86.9%, n = 40) diaphragm thickness in the standing position compared to supine. The same dependence, although to a lesser extent, also concerned diaphragm function indexes. Standing worsening the DTF index outcomes was shown at 63% (n = 29) in the left and at 56.5% (n = 26) in the right diaphragm dome, while left and right DTR decreases were manifested among 31 (67.3%) and 33 (71.7%) patients, respectively.

Only a small number of subjects had left (n = 6, 13%), right (n = 6, 13%), and both-sided (n = 3, 6.5%) diaphragm muscle paralysis. We detected 73.9% (n = 34) of patients with left side, 76% (n = 35) with right side, and 69.5% (n = 32) with simultaneous both side diaphragm atrophy. We noted a slightly greater, but not significant, number of individuals with diaphragm atrophy among males than females (75%, n = 15 vs. 65%, n = 17; *p* = 0.482), as well as in patients who did not use oxygen therapy over the COVID-19 course (77%, n = 20 vs. 60%, n = 12; *p* = 0.216). A particularly high percentage (*p* = 0.205) of this type of diaphragm dysfunction was reported in subjects with normal weight (90.9%, n = 10), determined by BMI, vs. overweight (64.7%, n = 11) and obesity (61.1%, n = 11). Similar findings were demonstrated in relation to age, where the youngest patient group, aged between 41 and 50 years, was characterized by the highest number (88.8%, n = 8) of diaphragm muscle mass thinning, compared to other age groups (51–60 years: 64.7%, n = 11; 61–70 years: 61.5%, n = 8; *p* = 0.654).

**Figure 2:**
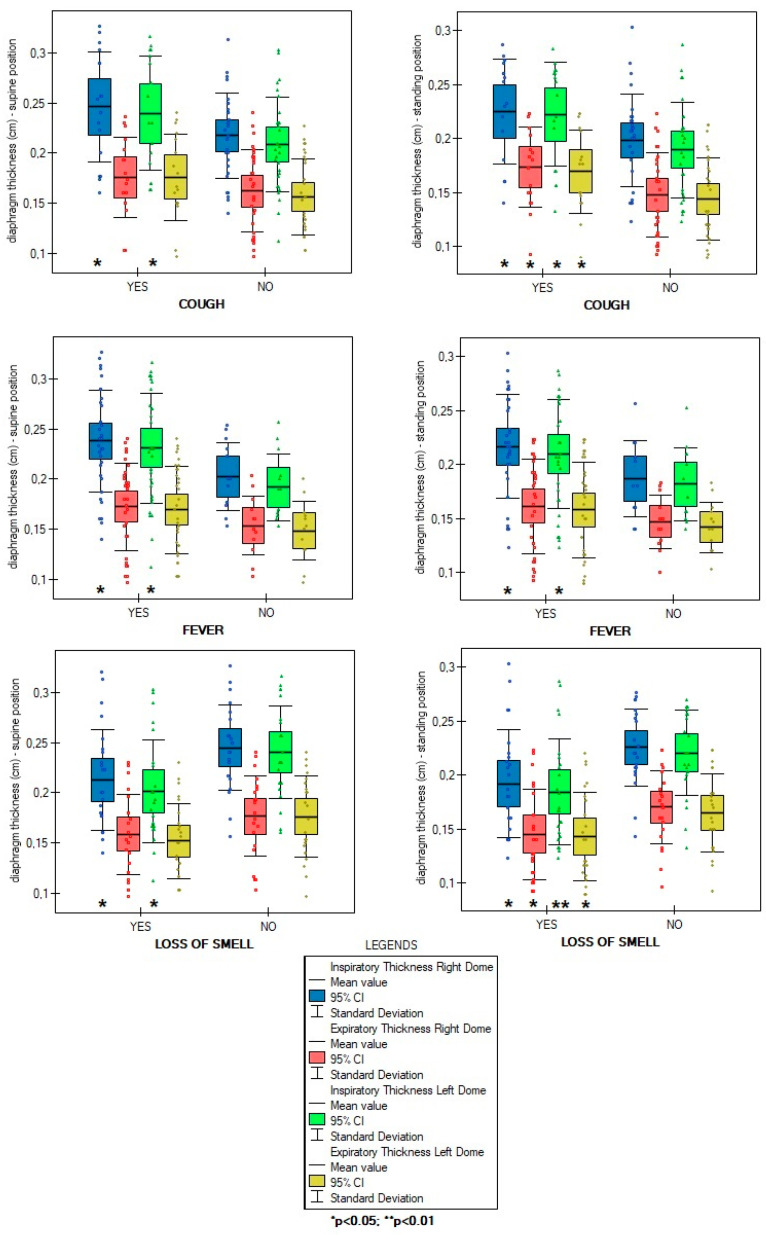
Differences in diaphragm inspiratory and expiratory thickness due to the presence of coughing, fever, and loss of smell in supine and standing positions.

**Figure 3:**
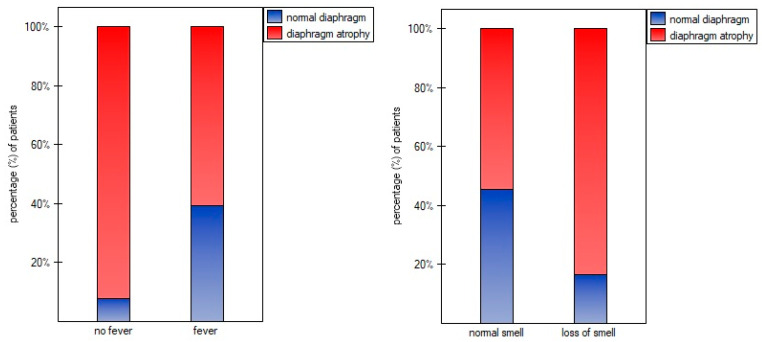
Percentage of patients with atrophy due to the presence of fever and loss of smell.

The overall number of symptoms during infection was not correlated with diaphragm function parameters in supine (ThIns: r = 0.003, *p* = 0.981; ThEx: r = 0.104, *p* = 0.488; DTF: r = −0.143, *p* = 0.345; DTR: r = −0.144, *p* = 0.338) and standing positions (ThIns: r = −0.023, *p* = 0.8798; ThExp: r = 0.072, *p* = 0.631; DTF: r = −0.140, *p* = 0.357; DTR: r = −0.144, *p* = 0.338). Of all analyzed disease symptoms, only patients with the presence of cough, fever, and without loss of smell over the COVID-19 course had significantly greater values of diaphragm inspiratory thickness in supine position, as well as greater inspiratory and expiratory thickness in standing position, compared to participants without the abovementioned symptoms (Figure 2). Furthermore, as shown in Figure 3, we found a significantly lower percentage of diaphragm atrophy among participants with fever (*p* = 0.0353) and without smell disorders (0.0340).

In further analysis, we compared diaphragm parameters’ relation to a number of statistically significant COVID-19 symptoms that occurred among patients during the disease. For this purpose, we divided participants into 4 categories: subgroup I (presence of only 1 symptom: cough, fever, or loss of smell; n = 16, 34.8%), subgroup II (presence of 2 from 3 symptoms; n = 24, 52.2%), subgroup III (all 3 symptoms presented; n = 3), and subgroup IV (none of the 3 symptoms reported; n = 3, 6.5%). Due to the small sample size of subgroups III and IV, we compared diaphragm parameters only between subgroups I and II. We found no differences, neither in the case of inspiratory (supine: *p* = 0.622; standing: *p* = 0.723) and expiratory thickness (supine: *p* = 0.489; standing: *p* = 0.648), nor in the case of the DTF (supine: *p* = 0.431; standing: *p* = 0.781) and DTR indexes (supine: *p* = 0.422; standing: *p* = 0.657). In the case of atrophy, the simultaneous presence of fever and smell loss was also not more associated with a lower risk of diaphragm atrophy than single-symptom occurrence (*p* = 0.4802).

**Figure 4:**
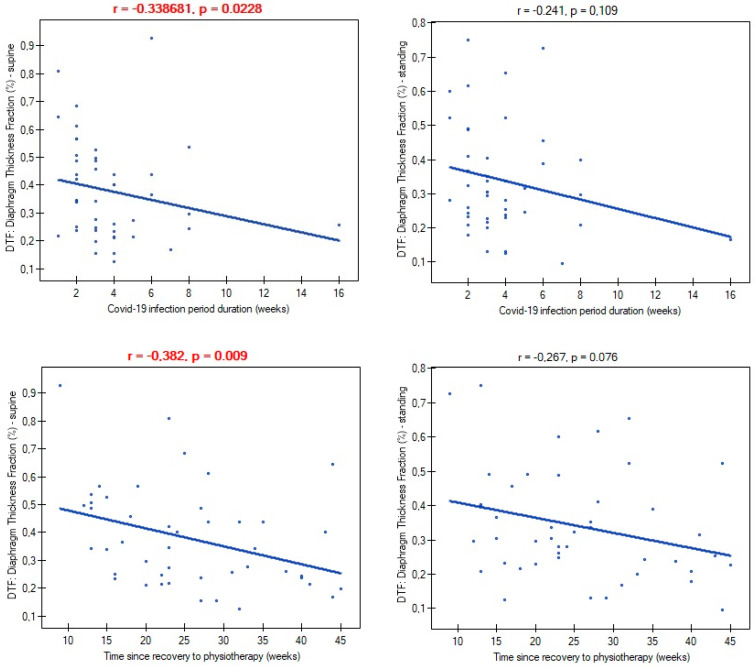
Correlation between time variables and the diaphragm thickness fraction index.

Mild and moderate severity of COVID-19 disease did not differentiate participants in terms of atrophy risk (mild: 66.6%; moderate: 70.9%; *p* = 0.782), supine diaphragm ultrasound measurements (ThIns: *p* = 0.684; ThExp: *p* = 0.956; DTF: *p* = 0.727; DTR: *p* = 0.482), and standing diaphragm ultrasound measurements (ThIns: *p* = 0.597; ThExp: *p* = 0.924; DTF: *p* = 0.365; DTR: *p* = 0.424). There were also no relationships between time of infection duration and diaphragm thickness in both supine (ThIns: r = 0.022, *p* = 0.880; ThExp: r = 0.151, *p* = 0.314) and standing positions (ThIns: r = −0.087, *p* = 0.563; ThExp: r = 0.002, *p* = 0.986). Likewise, time since recovery showed no association with muscle thickness at supine (ThIns: r = −0.167, *p* = 0.266; ThExp: r = 0.019, *p* = 0.899) and standing (ThIns: r = −0.190, *p* = 0.205; ThExp: r = −0.056, *p* = 0.707). In contrast, both variables had an impact on values of the DTF index (Figure 4).

For further analysis of the time variables, we split the data into a lower quartile (Q1), median quartile (Q2), and upper quartile (Q3). We found no differences in the proportion of diaphragm atrophy due to the time of infection duration (*p* = 0.810): <2 weeks (75%, n = 12), 3–4 weeks (65%%, n = 13), and over 4 weeks (70%, n = 7), as well as the time since recovery to start physiotherapy (*p* = 0.713): <17 weeks (61.5%, n = 9), 18–32 weeks (75%, n = 15), and over 32 weeks (61.5%, n = 8).

**Figure 5:**
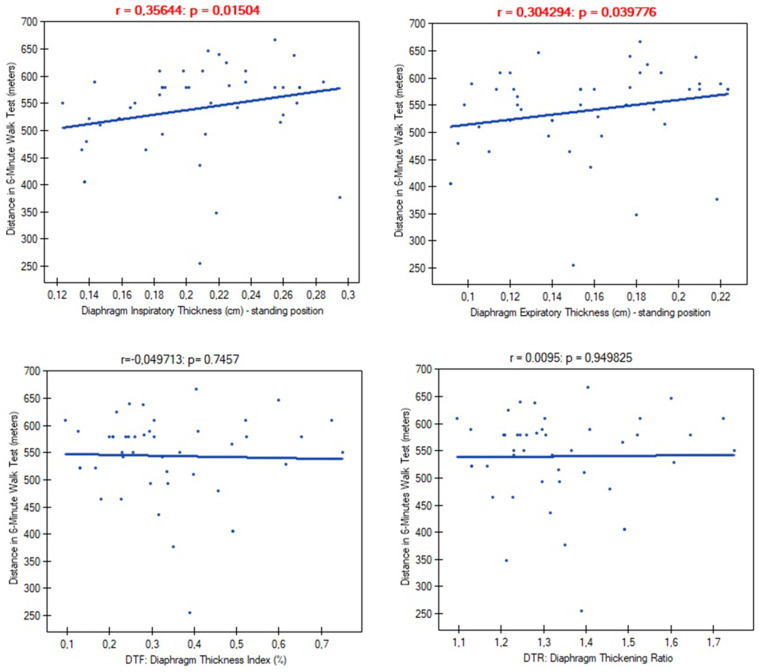
Correlation between diaphragm functional parameters and distance in the 6-min walk test.

In further analysis, we examined links between diaphragm functional parameters and participants’ exercise tolerance. We found significant correlations of diaphragm muscle thickness with the distance obtained in the 6-min walk test. However, there were no significant differences in the case of DTR and DTF indexes (Figure 5).

The 6-MWT-derived variables, such as the saturation level before and after the test, the exertion and dyspnea levels measured on the Borg scale, and the Metabolic Equivalent Task also showed dependence on the diaphragm muscle mass (Table 3).

**Figure 6:**
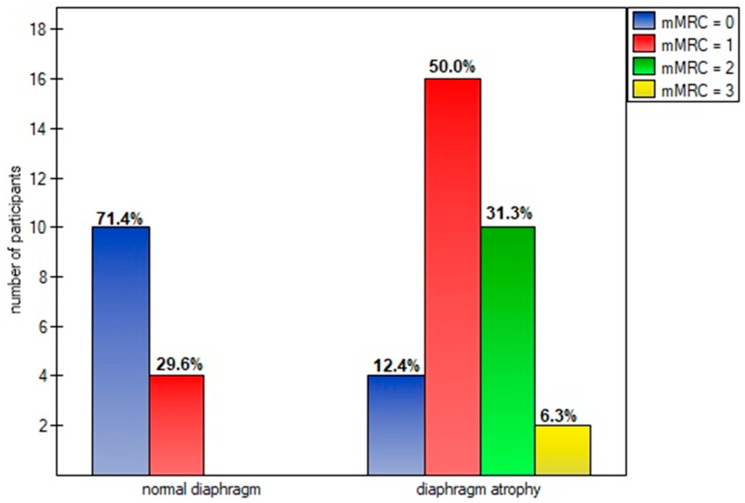
Percentage of diaphragm atrophy due to points obtained on the mMRC scale.

**Figure 7:**
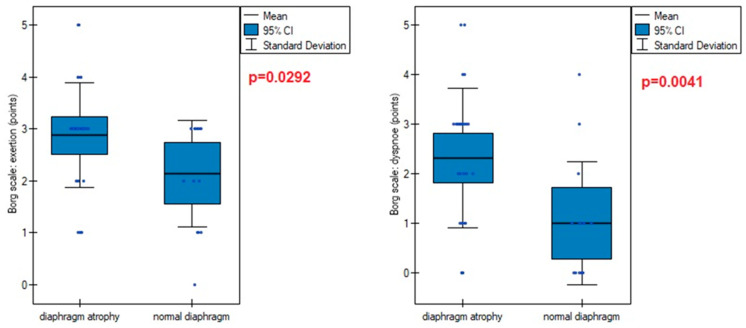
Points obtained on the Borg subscales (exertion and fatigue) in patients with normal and atrophic diaphragm.

Diaphragm muscle atrophy strongly contributed (*p* = 0.0005) to functional dyspnea during activities of daily living, measured on the mMRC scale (Figure 6). This was also evident in the higher points obtained on the Borg scale in participants with an atrophic diaphragm compared to subjects without dysfunction (Figure 7).

However, there were no differences in the saturation before the test (95.30 vs. 95.00; *p* = 0.5910), saturation after the test (95.68 vs. 94.78, *p* = 0.1411), MET (7.16 vs. 7.81; *p* = 0.2956), and 6-min walk test distance (530.71 vs. 559.92; *p* = 0.2786).

## 4. Discussion

Our study achieved the intended goals. The main finding presented here was the high percentage of patients with diaphragm muscle function disorders. The most prevalent was diaphragm atrophy, which was present in 75% of participants. The occurrence of this type of dysfunction was strongly associated with worse physical functional capacity. We also identified several factors that increase the risk of, as well as prevent, atrophy.

According to recent literature, the pathophysiology of COVID-19 infection is not limited to the lungs but causes a cascade of systemic events, affecting various organs and tissues. To date, several studies have tried to determine the impact of SARS-CoV-2 on the diaphragm muscle. Imamovic et al. described an incidence of diaphragmatic rupture as a late consequence of SARS-CoV-2 infection [28]. Shi et al. demonstrated distinct myopathic changes in the diaphragm muscle specimens of deceased COVID-19 patients. Autopsies from 26 patients showed an increased expression of angiotensin-I-converting enzyme 2 (ACE2) in the diaphragm (predominantly localized at the myofiber membrane), as well as a two-fold higher degree of epimysial and perimysial fibrosis in the postmortem diaphragms of COVID-19 patients admitted to the ICU compared with the non-COVID-19 ICU control group, with comparable durations of mechanical ventilation and ICU length of stay. The authors concluded that these histological changes may impact diaphragm contractility and contribute to the chronic sensation of dyspnea and fatigue [29]. However, it is unclear whether such diaphragm tissue changes are present in SARS-CoV-2 survivors. Indirect confirmations of this assumption are contained in other studies. Deboer and colleagues found an inverse correlation between diaphragm muscle strength and breathlessness after recovery, which may point to diaphragm fibrosis [30]. Formenti et al. demonstrated a significantly lower echogenicity of the diaphragm muscle on ultrasound among patients with acute respiratory distress syndrome arising from COVID-19 compared to non-survivors [31]. Not just structural, but also functional diaphragm disorders were reported in the literature. Several studies demonstrated cases of unilateral or/and bilateral diaphragm paralysis due to active SARS-CoV-2 infection [32,33,34,35]. Borroni described two cases of diaphragmatic myoclonus as a neurological manifestation of SARS-CoV-2 infection without evidence of structural damage of the central nervous system [36]. Satici et al. detected diaphragm elevation among patients with persistent dyspnea without lung parenchymal involvement, who had recovered from COVID-19, as well as decreased diaphragm excursion in most patients [37]. Other researchers reported that diaphragmatic movement predicted successful weaning in patients with COVID-19. The study included 22 patients with severe COVID-19 who were invasively mechanically ventilated for 2 days or more. A patient with diaphragmatic excursion > 12 mm during the spontaneous breathing trial is unlikely to be re-intubated [38]. Umbrello et al. observed that both diaphragm sizes were significantly reduced at day 7 from ICU admission in COVID-19 patients. A significantly greater reduction was reported in non-survivors compared to survivors [39]. Corradi et al. identified a low diaphragm thickness fraction index as a predictor of Continuous Positive Airway Pressure (CPAP) failure, as its values were inversely correlated with its success (the lower the DTF values, the more likely the CPAP failure) [40]. In another study, the same authors examined 77 patients who underwent diaphragm ultrasonography within 36 h of admission. They found that a low diaphragm thickness was an independent predictor of adverse outcomes in COVID-19 patients, with the end expiratory diaphragm thickness being the strongest [41]. Similarly, Van Steveninck et al. described the case of a low value of diaphragm thickness prior to patient intubation, while respiratory failure was not yet evident from arterial blood gas analysis in patients with COVID-19 pneumonia [42].

The results of our study should add new knowledge to this field and should contribute to an even better understanding of the pathophysiology and treatment of respiratory muscle pathology after SARS-CoV-2 infection. Data contained in Table 2 indicate a low inspiratory and expiratory diaphragm thickness, as well as a very low diaphragm thickness fraction (DTF). Importantly, patients presented worse outcomes in the standing position compared to the supine position, where the abdominal wall is relaxed and resistance from visceral organs is lower. This suggests an impairment of diaphragm muscle contraction force and its ability to work properly during physical effort. The best illustration of the dysfunction scale can be seen by comparing the results obtained here with the values of healthy subjects, where the normal percentage of thickening was 65% in supine (36% in our study) and 174% in standing (32% in our study) [20]. This poor diaphragm contractility was strongly linked with parameters of physical functional capacity (Table 3). The low inspiratory and expiratory diaphragm thickness in standing position corresponded with the shorter distance obtained by participants during the 6-min walk test. Additionally, patients with low DTF index values demonstrated a higher level of dyspnea and exertion on the Borg scale after the test. Values of the DTF index were also strongly negatively correlated with low oxygen saturation levels measured after the test. We found no association of the DTF index with resting oxygenation. This indicates that, despite its thinning, the diaphragm fulfills its primary function of ensuring gas exchange at rest, but after the infection, it is too weak to perform the optimal ventilation process during physical exertion.

In our study, almost 85% of post-COVID-19 patients that qualified for the physiotherapy program had at least one sonographic abnormality of diaphragm muscle function. These finding are consistent with a previous study conducted by Farr et al. [43]. The vast majority of these dysfunctions were atrophy (69.5%), unlike the other viral diseases described in the literature that caused diaphragm paralysis [5,6,7,8,9,10,11]. The impact of diaphragm muscle thinning was particularly evident in the degree of disability that breathlessness posed on day-to-day activities. A significantly higher percentage of patients classified their objectively perceived respiratory disability in the first, second, or third class of dyspnea on the mMRC scale, compared to patients with a normal muscle cross-section (Figure 5). The reduction in diaphragm muscle mass also resulted in higher exertion and dyspnea after the 6-min walk test compared to non-atrophic patients.

In our study, we determined several factors that significantly contributed to diaphragm dysfunction or prevented its occurrence. Patients who experienced cough, fever, and normal olfaction over the course of infection had greater inspiratory muscle thickness compared to those without these symptoms (Figure 2). Fever and normal olfaction also played a protective role against diaphragm atrophy (Figure 3). However, patients who reported the presence of two out of the three symptoms (cough, fever, and lack of smell) did not have greater inspiratory and expiratory thickness than those with only one of these symptoms. Similarly, the simultaneous presence of fever and the lack of smell disorders were not associated with lower rates of atrophy than the presence of one symptom. This suggests that the occurrence of just one of these factors should be enough to ensure the proper functioning of the main respiratory muscle and could potentially protect against muscle mass loss over the course of COVID-19 infection. The explanation for the influence of these factors should be sought in human physiology. The diaphragm muscle is involved in non-ventilatory expulsive behaviors. The cough mechanism causes diaphragm contraction; therefore, frequent coughing during infection seems to prevent muscle thinning by stimulating it to contract. In the case of fever, a higher respiratory rate per minute seems to be significant. It is much more difficult to explain the relationship between smell disorders and reduced diaphragm muscle mass. Previous studies reported that odor stimuli change respiratory patterns. Unpleasant odors decrease tidal volume and increase respiratory frequency, resulting in a rapid and shallow breathing pattern. We suppose that anosmia over the COVID-19 course could have caused similar changes in respiratory rhythm, thereby causing a decrease in diaphragm muscle mass.

We demonstrated the presence of diaphragm atrophy in the mild and moderate stages of COVID-19. Previous studies have shown the presence of this type of diaphragm dysfunction in people with a severe and critical course of the disease [43]. This allowed us to conclude that atrophy occurred independently of infection severity, except in asymptomatic patients. The duration of infection had no impact on inspiratory and expiratory diaphragm thickness, as well as the percentage of atrophy diagnoses. However, a longer duration of infection was associated with greater impairment of diaphragm contractility, as measured by the DTF index. A similar relationship was noted between time since recovery and the magnitude of diaphragm contractility. Considering that our patients had no previous physiotherapy, as well as the time from recovery to the start of physiotherapy (mean: 22 weeks) was negatively correlated with the DTF index, we can state that it is not a temporary dysfunction that resolves spontaneously over time, but rather it turns into a chronic problem, with a visible tendency to aggravate the problem in the absence of physiotherapy. Breathing alone seems to be insufficient to regain the decreased muscle mass.

Sonographic evaluation of the diaphragm muscle has gained popularity due to its non-invasive nature and the ability to assess diaphragmatic function at the bedside in various clinical conditions. In post-COVID-19 patients, diaphragm ultrasound can be utilized to diagnose potential diaphragm dysfunctions, as well as to monitor the efficacy and follow-up of pulmonary physiotherapy. The goal of breathing therapy in this patient group should be to regain diaphragm muscle mass and improve their strength. It seems that ultrasound can also be used during the physiotherapy process as a biofeedback tool to achieve an effective diaphragmatic breathing pattern.

The strength of our study lies in the comprehensive ultrasound assessment of diaphragm muscle function and its correlation with many clinical variables over the course of COVID-19, allowing us to obtain a broad clinical picture of this problem. We considered that many patients experience dyspnea and fatigue during less or more intensive physical activity. To obtain the most reliable results, we performed ultrasound assessment not only in the supine position but also in the standing position. However, our study has a few limitations. Despite measurements in two positions, we were not able to detect the percentage of atrophy and paralysis of the diaphragm in the standing position. This was due to a gap in the medical literature, where a cutoff value for detecting atrophy has only been described in reference to the supine position. In our opinion, this did not affect the results of the research, as the percentage of atrophy may be higher and the correlations even more statistically significant. Another limitation was the absence of a well-matched control group consisting of participants who did not perceive breathing difficulties after COVID-19. Unfortunately, these patients were not qualified for the physiotherapy program in our ward. Some patients were not enrolled in the final study group due to the presence of exclusion criteria. Thus, the small sample size should lead to caution in interpreting the results. Moreover, the retrospective analysis of symptoms over the COVID-19 course may have led to the omission of some information. Finally, respiratory patterns, gas exchange, and pulmonary function were not analyzed, and physical capacity was only assessed indirectly. Therefore, our findings that diaphragm atrophy may be a potential factor contributing to reduced physical capacity should be carefully considered, given the limitations of our estimation.

## 5. Conclusions

In conclusion, diaphragm muscle dysfunction is a very common and serious long-term post-COVID-19 consequence, contributing to prolonged functional impairments. Pulmonary rehabilitation can lead to improvements in respiratory function and should be implemented at an early recovery stage.

## Figures and Tables

**Table 1:** Characteristics of the study population (N = 46).

Variables	M ± SD [95% CI] or N (%)
Age (years):	58.24 ± 10.70 [55.02–61.46]
<40 years	2 (4.44%)
41–50 years	9 (20.0%)
51–60 years	16 (35.55)
61–70 years	13 (28.88%)
>70 years	5 (11.11%)
Gender:	
male	20 (43.47%)
female	26 (56.52%)
Height (cm)	1.67 ± 0.08 [1.64–1.69]
Weight (kg)	79.64 ± 16.50 [74.73–84.54]
BMI (points):	28.42 ± 4.83 [26.98–29.85]
18.50–24.99 (normal weight)	11 (23.91%)
25.00–29.99 (overweight)	17 (36.95%)
>30.00 (obesity)	23 (50.0%)
COVID-19 symptoms:	
lack of symptoms	0 (0.0%)
cough	29 (63.04%)
fever	33 (71.73%)
dyspnea	33 (71.73%)
loss of smell	24 (52.17%)
loss of taste	23 (50.0%)
chest pain	20 (43.47%)
neck pain	8 (17.39%)
vomiting	5 (10.87%)
reflux	1 (2.17%)
general muscle pain	30 (65.21%)
intercostal pain	10 (21.73%)
mean number of symptoms per patient	4.80 ± 1.83 [4.25–5.34]
COVID-19 symptom duration time (weeks)	3.76 ± 2.59 [2.99–4.53]
Time since recovery to start physiotherapy (weeks)	26.65 ± 9.98 [22.68–28.61]
Severity of COVID-19 disease:	
asymptomatic	0 (0%)
mild	12 (26.09%)
moderate	31 (67.39%)
severe	3 (6.52%)
critical	0 (0%)
Oxygen therapy usage during COVID-19 course:	
yes	21 (45.65%)
no	25 (54.35%)
mMRC scale (points):	1.24 ± 0.80 [1.00–1.48]
grade 0	14 (30.43%)
grade 1	20 (43.48%)
grade 2	10 (21.74%)
grade 3	2 (4.35%)
6-min walk test distance (meters)	539.60 ± 83.29 [514.87–564.34]
Borg scale (points):	
fatigue	2.65 ± 1.05 [2.33–2.96]
dyspnea	1.91 ± 1.47 [1.47–2.35]
Saturation:	
before 6-min walk test	95.51 + 1.78 [94.68–95.74] 95.41 + 1.90 [94.84–95.97]
after 6-min walk test	
MET (mL O_2_/min/kg)	7.35 ±1.92 [6.78–7.93]

Abbreviations. BMI: body mass index; mMRC: Modified Medical Research Council; MET: Metabolic Equivalent Task. Notes. Values are presented as means ± standard deviation [95% confidence interval]. Number of subjects: 46.

**Table 2:** Descriptive statistics of diaphragm functional parameters.

Variables		Supine Position	Standing Position	*p*-Value
Diaphragm Inspiratory Thickness (cm)	left	0.219 ± 0.052 (0.204–0.235)	0.201 ± 0.047 (0.187–0.216)	**<0.001**
	right	0.228 ± 0.049 (0.213–0.242)	0.208 ± 0.046 (0.194–0.222)	**<0.001**
	*p*-value	0.085	0.089	-
Diaphragm Expiratory Thickness (cm)	left	0.163 ± 0.040 (0.151–0.175)	0.153 ± 0.039 (0.141–0.165)	0.121
	right	0.167 ± 0.040 (0.155–0.179)	0.157 ± 0.039 (0.145–0.169)	0.113
	*p*-value	0.121	0.093	-
Diaphragm Thickness Fraction (DTF) (%)	left	36.26 ± 18.72 (30.70–41.82)	33.03 ± 15.77 (28.34–37.71)	0.118
	right	38.69 ± 18.99 (33.05–44.33)	34.42 ± 18.30 (28.98–39.85)	0.087
	*p*-value	0.159	0.439	-
Diaphragm Thickening Ratio (DTR)	left	1.367 ± 0.186 (1.311–1.423)	1.332 ± 0.158 (1.284–1.380)	0.096
	right	1.390 ± 0.190 (1.332–1.447)	1.347 ± 0.183 (1.292–1.402)	0.093
	*p*-value	0.197	0.420	-

Notes. Number of subjects: 46. Results are presented as means ± standard deviation (95% confidence interval). Student’s *t*-test was used to compare groups.

**Table 3:** Correlation between diaphragm function parameters and selected clinical variables (N = 46).

Variables		Saturation before 6-Min Walk Test	Saturation after 6-Min Walk Test	Borg Scale: Fatigue	Borg Scale: Dyspnea	MET
ThIns	r	−0.021	−0.020	**−0.534**	**−0.417**	**0.323**
	*p*-value	0.8848	0.8914	**0.0001**	**0.0038**	**0.0281**
ThExp	r	−0.083	−0.128	**−0.362**	**−0.302**	0.277
	*p*-value	0.5814	0.3959	**0.0134**	**0.0409**	0.0615
DTF	r	0.171	**0.334**	−0.129	−0.077	−0.0541
	*p*-value	0.2598	**0.0248**	0.3954	0.6117	0.7239
DTR	r	0.163	**0.324**	−0.138	−0.098	−0.013
	*p*-value	0.2782	**0.0280**	0.3596	0.5129	0.9290

Notes. ThIns: inspiratory thickness, ThExp: expiratory thickness, DTF: diaphragm thickness fraction, and DTR: diaphragm thickening ratio. Number of subjects: 46. Correlations analysis was performed using Spearman’s correlation test. Bold express statistically significant correlations.

## Data Availability

The data presented in this study are available upon request from the corresponding author.
